# The Prognostic Significance of the DBIL/HDLC Ratio in Patients With Dilated Cardiomyopathy

**DOI:** 10.1155/cdr/8835736

**Published:** 2025-02-15

**Authors:** Xinyi Wang, Qiqi Song, Qingqing Zhang, Xinyi Li, Jiaqi Wang, Jiantao Gong, Ziyi Zhang, Ning Tan, Suk-Ying Tsang, Wing-tak Wong, Dunliang Ma, Lei Jiang

**Affiliations:** ^1^Department of Cardiology, Guangdong Cardiovascular Institute, Guangdong Provincial People's Hospital, Guangdong Academy of Medical Sciences, Guangzhou, China; ^2^School of Medicine, South China University of Technology, Guangzhou, China; ^3^Department of Anesthesiology, Guangdong Provincial People's Hospital Ganzhou Hospital, Ganzhou Municipal Hospital, Ganzhou, China; ^4^School of Life Sciences, The Chinese University of Hong Kong, Hong Kong SAR, China; ^5^Department of Applied Biology and Chemical Technology, The Hong Kong Polytechnic University, Hong Kong, China

**Keywords:** DBIL, dilated cardiomyopathy, HDLC, prognosis

## Abstract

**Background:** In cardiovascular pathology, both direct bilirubin (DBIL) and high-density lipoprotein cholesterol (HDLC) have been associated with adverse clinical outcomes. However, the prognostic significance of these biomarkers in the context of dilated cardiomyopathy (DCM) remains unclear. To address this gap, this study conducted a retrospective analysis to evaluate the prognostic value of the DBIL/HDLC ratio in patients diagnosed with DCM.

**Methods and Results:** A total of 986 consecutive DCM patients were retrospectively enrolled from January 2010 to December 2019 and divided into two groups based on the DBIL/HDLC ratio cut-off value: ≤ 4.45 (*n* = 483) and > 4.45 (*n* = 503). Patients with lower DBIL/HDLC (≤ 4.45) experienced lower in-hospital mortality, long-term mortality, and major adverse clinical events (MACEs) (0.8%, 32.9%, and 12.2%, respectively) compared to those with higher DBIL/HDLC (> 4.45) (6.4%, 59.1%, and 16.7%, respectively). Multivariate analysis identified DBIL/HDLC as an independent risk factor for long-term mortality (odds ratio: 1.026; 95% confidence interval (CI): 1.005–1.048; *p* = 0.016) and all-cause mortality over a median follow-up of 67 ± 1.8 months (hazard ratio: 1.011; 95% CI: 1.005–1.018; *p* < 0.001). The receiver operating characteristic curve showed good discrimination for long-term mortality (area under the curve (AUC): 0.675; 95% CI: 0.692–0.708; *p* < 0.001). The Kaplan–Meier survival analysis demonstrated a better prognosis for patients with DBIL/HDLC ≤ 4.45 (log-rank *χ*^2^ = 40.356, *p* < 0.001). Furthermore, the impact of additional variables on the results was investigated by a subgroup analysis.

**Conclusion:** The DBIL/HDLC ratio could serve as a simple and cost-effective tool for evaluating prognosis in DCM.

## 1. Introduction

Dilated cardiomyopathy (DCM), characterized by left ventricular enlargement and systolic dysfunction (left ventricular ejection fraction (LVEF) < 45%) in the absence of identifiable causes other than genetic predispositions, presents significant diagnostic and therapeutic challenges [1]. As a predominant form of cardiomyopathy, DCM has a high prevalence, affecting approximately 1 in 250–500 individuals [2]. The etiology of DCM can be categorized into genetic (primary DCM) and acquired (secondary DCM) forms [3]. Approximately 50% of cases have a discernible etiology, while the remaining cases are classified as idiopathic DCM [4]. Previous research, including our own, has elucidated the pathogenesis of DCM through genetic and molecular studies [5]. Despite advancements in molecular and genetic diagnostics that have facilitated the development of gene-specific interventions, the high costs and limited accessibility within certain healthcare systems severely limit their application [6]. Current therapeutic strategies for DCM include the use of angiotensin-converting enzyme inhibitors or angiotensin receptor blockers, *β*-blockers, mineralocorticoid receptor antagonists, and sodium-glucose cotransporter 2 (SGLT2) inhibitors [7]. However, DCM patients remain at risk for acute episodes of decompensated heart failure (HF), malignant arrhythmias, or sudden death, underscoring the need for accurate prognostic indicators to predict clinical outcomes [8].

Alterations in systemic hemodynamics resulting from hepatic impairment influence both diastolic and systolic cardiac function through changes in atrial blood volumes [9]. In actual clinical practice, elevated levels of direct bilirubin (DBIL) are typically indicative of significant, irreversible hepatic injury [10]. However, clinical and epidemiological studies have also implicated bilirubin in the pathogenesis of cardiometabolic diseases [11]. A Chinese cohort, the Dongfeng-Tongji cohort, identified that DBIL levels were independently associated with an elevated risk of coronary heart disease (CHD) incidence [12]. In practical clinical application, decreased high-density lipoprotein cholesterol (HDLC) levels indicate metabolic dyslipidemia, which is associated with a higher risk of cardiovascular disease (CVD) outcomes [13]. Another clinical trial has demonstrated that patients who received Danshen (*Salvia miltiorrhiza*) compounds showed lower DBIL levels and higher HDLC levels, which reduced the risk of CHD [14]. The opposing trends of DBIL and HDLC were also observed in alcoholic liver cirrhosis (ALC) [15]. Thus, it is necessary to explore whether the DBIL/HDLC ratio could be a meaningful predictor for CVD. Xie et al. proposed a model in which HDLC is one of the largest contributors to the early differentiation of left ventricular reverse remodeling (LVRR) and non-LVRR in patients with newly diagnosed DCM [16]. Given the complexity of this model due to its numerous variables, we suggest that the DBIL/HDLC ratio may serve as a simpler tool for assessing prognosis in patients with DCM.

## 2. Methods

### 2.1. Study Population

**Figure 1:**
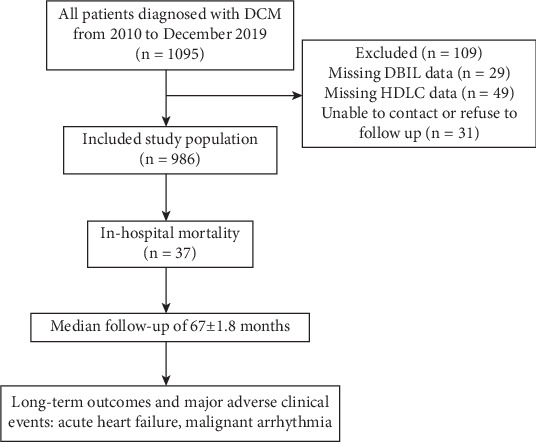
The criteria of inclusion and exclusion.

A retrospective study was conducted at the Guangdong Provincial People's Hospital, Guangzhou, China, involving 1095 consecutive DCM patients from January 2010 to December 2019. Patients were diagnosed according to the American Heart Association's criteria [17]. DBIL data were missing in 29 patients, and HDLC data were missing in 49 patients; 31 patients were lost to follow-up. Consequently, 986 patients were included in the retrospective study. The ethics committee of Guangdong Provincial People's Hospital approved this study, granting a waiver for written informed consent due to its retrospective nature. Oral consent was obtained from patients or their relatives by telephone during follow-up (Figure 1).

### 2.2. Data Extraction

Data on baseline characteristics, medical history, and laboratory findings were retrieved from electronic medical records. Clinical data, including demographics, comorbidities, complete blood counts, biochemical profiles, and New York Heart Association (NYHA) classifications, were meticulously collected and verified. Patient outcomes were monitored from admission until the conclusion of the study, with survival status and severe diagnoses recorded.

### 2.3. Study Endpoints

The primary endpoint was in-hospital mortality, while the secondary endpoints were long-term mortality and major adverse clinical events (MACEs), which included acute heart failure (AHF) and malignant arrhythmia but not vascular diseases. Follow-up was conducted via telephone interviews or a review of outpatient records in 2021.

### 2.4. Statistical Analysis

Data analysis was conducted using SPSS software, Version 25.0 (IBM Corp., Armonk, NY, United States). A two-sided *p* value of less than 0.05 was considered indicative of statistical significance. To maintain analytical integrity, records with missing values were excluded from the analysis. The DBIL/HDLC ratio was treated as a continuous variable for analysis, with continuous variables reported as mean ± standard deviation (SD).

For variables that did not follow a normal distribution, the Mann–Whitney *U* test was used for group comparisons. To assess survival outcomes, the Kaplan–Meier curves were generated, and the differences between groups were analyzed using the log-rank test. The utility of the DBIL/HDLC ratio in predicting adverse clinical events was quantified through receiver operating characteristic (ROC) curve analysis, which also helped establish the optimal cut-off value. Both univariate and multivariate logistic regression analyses, along with Cox proportional hazards models, were employed to explore the relationship between the DBIL/HDLC ratio and clinical outcomes. Variables that demonstrated significance in univariate analyses—excluding those related to the DBIL/HDLC ratio—were further examined in the multivariate models to ascertain their independent predictive value. To assess whether the prognostic effect of the DBIL/HDLC ratio for DCM patients depends on baseline characteristics, we divided the patients into the following subgroups to evaluate its prognostic value: age (≤ 70, > 70), sex (male, female), and LVEF (≤ 40%, > 40%).

## 3. Result

### 3.1. Baseline Characteristics

Among the cohort of 986 patients (643 males and 343 females) who met the inclusion criteria, individuals were categorized based on their long-term clinical outcomes. The baseline characteristics are summarized in Table 1. Analysis revealed that deceased participants tended to be older, with elevated levels of glucose, creatinine (CREA), and DBIL, along with reduced serum chloride and HDLC levels. Furthermore, the incidence of decreased cardiac function (according to NYHA functional class) was notably higher in the deceased cohort (Table 1).

**Figure 2:**
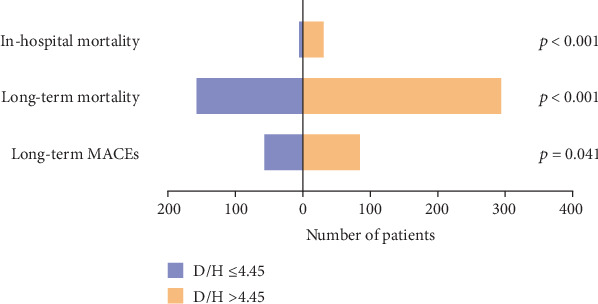
The prevalence of in-hospital and long-term clinical outcomes in patients. D/H means DBIL/HDLC ratio.

Hospital mortality was observed in 37 patients (3.7%). The incidence of in-hospital mortality increased with a rising DBIL/HDLC ratio, from 0.8% in patients with a ratio ≤ 4.45 to 6.4% in those with a ratio > 4.45 (*p* < 0.001). Over a median follow-up duration of 67 ± 1.8 months, 456 patients died, and 27 were lost to follow-up. The occurrences of MACEs (12.2% vs. 16.7%) and long-term mortality (32.9% vs. 59.1%) were significantly higher in patients with elevated DBIL/HDLC ratios (Figure 2).

### 3.2. Predictive Value of the DBIL/HDLC Ratio for In-Hospital Mortality

Univariate logistic regression analysis identified the DBIL/HDLC ratio as a significant predictor of in-hospital mortality (odds ratio (OR): 1.030; 95% confidence interval (CI): 1.017–1.043; *p* < 0.001) (Table 2). When accounting for variables that showed significant differences between survivors and nonsurvivors, such as serum CREA, log-transformed N-terminal B-type natriuretic peptide (lgNT-proBNP), and LVEF, the DBIL/HDLC ratio continued to significantly predict in-hospital mortality in the multivariate model (OR: 1.018; 95% CI: 1.004–1.032; *p* = 0.013).

**Figure 3:**
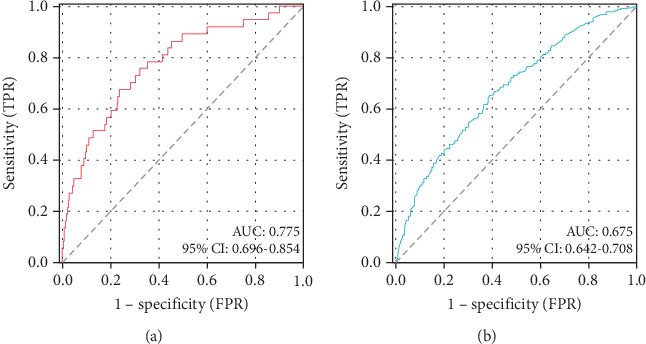
ROC curves of DBIL/HDLC ratio for predicting (a) in-hospital and (b) long-term mortality. The optimal cut-off value of the DBIL/HDLC ratio was 7.90 for predicting in-hospital mortality and 4.45 for predicting long-term mortality.

ROC curve analysis demonstrated the DBIL/HDLC ratio's strong predictive capacity for in-hospital mortality, with an area under the curve (AUC) of 0.775 (95% CI: 0.696–0.854; *p* < 0.001). The identified optimal cut-off value was 7.90, yielding a sensitivity of 67.6% and a specificity of 76.4% (Figure 3(a)).

### 3.3. DBIL/HDLC Ratio in Predicting Long-Term Outcomes

Univariate logistic regression analysis found that the DBIL/HDLC ratio was related to long-term mortality (OR: 1.026; 95% CI: 1.005–1.048; *p* = 0.016) (Table 3). The follow-up period included a total of 456 all-cause deaths (299 males and 157 females). The DBIL/HDLC ratio also proved to be a significant predictor of long-term mortality. The optimal cut-off value of the DBIL/HDLC ratio for predicting long-term mortality (AUC: 0.675; 95% CI: 0.642–0.708; *p* < 0.001) (Figure 3(b)), with a sensitivity of 65.1% and specificity of 61.1%.

**Figure 4:**
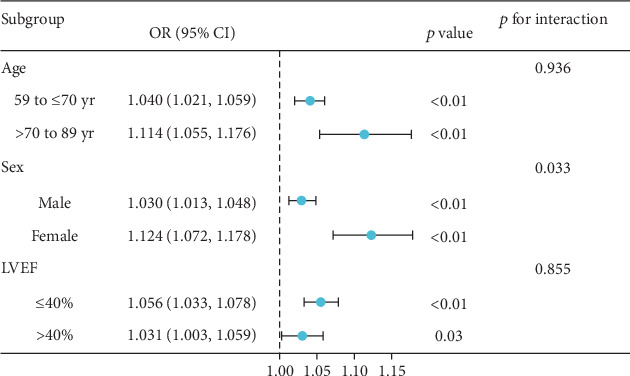
This forest plot presents the odds ratios (ORs) and 95% confidence intervals (CIs) for different subgroups based on their DBIL/HDLC ratio, with a cut-off point of 4.45.

Subgroup analyses were performed to assess whether the relationship between the DBIL/HDLC ratio and long-term mortality was influenced by baseline characteristics. It is well known that age, gender, and LVEF impact the prognosis of HF [18]. We included age, gender, and LVEF as adjustment factors in the subgroup analysis for DCM patients. Considering that patients with an LVEF of 40% or less are classified as having heart failure with reduced ejection fraction (HFrEF) [19], the DCM patients were also divided into LVEF ≤ 40% and > 40% to evaluate the ORs and 95% CIs for different subgroups. As shown in Figure 4, the OR values of all subgroups were more than 1, indicating that the long-term mortality increased significantly with the increase of the DBIL/HDLC ratio. Overall, there was no significant interaction between most groups. However, we observed that age exhibited a significant interaction with sex (*p* for interaction = 0.033), potentially affecting the relationship between the DBIL/HDLC ratio and long-term mortality. Therefore, we reanalyzed the relationship between DBIL/HDLC and long-term mortality in different sex groups. Both males and females demonstrated an increased risk of long-term mortality with a higher DBIL/HDLC ratio.

**Figure 5:**
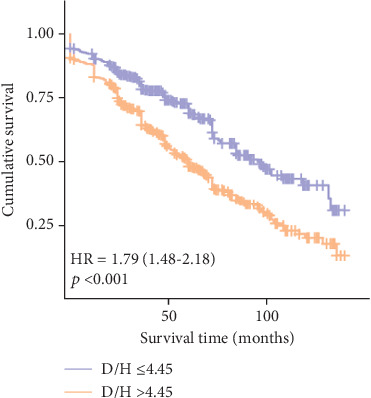
The Kaplan–Meier survival curves according to the cut-off value of DBIL/HDLC.

The Kaplan–Meier survival analysis revealed a significantly worse prognosis for patients with a DBIL/HDLC ratio > 4.45 compared to those with a ratio ≤ 4.45 (log-rank *χ*^2^ = 40.356, *p* < 0.001) (Figure 5). Furthermore, multivariate Cox proportional hazards analysis highlighted the DBIL/HDLC ratio as an effective predictor of mortality risk after adjusting for multiple variables (hazard ratio (HR): 1.011; 95% CI: 1.005–1.018; *p* < 0.001) (Table 4).

## 4. Discussion

This study explores the prognostic significance of the DBIL/HDLC ratio in patients with DCM. Our findings support the hypothesis that the DBIL/HDLC ratio serves as an independent prognostic indicator for patients with DCM. A lower DBIL/HDLC ratio was associated with better clinical outcomes.

DCM is characterized by progressive myocardial damage, leading to ventricular dilation and impaired cardiac function [3]. The risk of AHF and malignant arrhythmias increases with DCM progression, significantly elevating the risk of all-cause mortality [20]. Through this study, we have demonstrated the predictive reliability and clinical utility of the DBIL/HDLC ratio in forecasting outcomes in elderly DCM patients, highlighting its potential as a valuable prognostic marker for this condition.

DBIL is the conventional liver function and has clinical implications as the standard index of liver disorder. An elevated level of DBIL may suggest damage to the hepatic cells [21]. The increased DBIL is correlated with heart disease. Wang et al. suggested that DBIL is an independent prognostic predictor for HFpEF (heart failure with preserved ejection fraction) [22]. Patients with heart disease typically exhibit elevated central venous pressure and reduced arterial perfusion due to decreased cardiac output, which can contribute to liver dysfunction [23]. From a pathophysiological perspective, elevated DBIL levels may reflect increased oxidative stress and an amplified inflammatory response, both of which contribute to cardiomyocyte damage and potentially accelerate the progression of DCM [24].

Many researches have demonstrated the protective role of HDLC in atherosclerotic cardiovascular disease (ASCVD) [25]. Abnormal HDLC levels typically indicate dysregulation of lipid metabolism. Decreased HDLC levels are frequently observed in patients with nonalcoholic fatty liver disease (NAFLD). Deprince, Haas, and Staels suggested that dysregulated lipid metabolism serves as a critical link between NAFLD and CVD [26]. The alterations in hepatic lipid metabolism induced by NAFLD may influence arterial wall infiltration and promote the development of atherosclerotic plaques. Thus, this process can contribute to damage in other organs and lead to adverse outcomes [27]. HDLC plays an antiatherosclerosis role through reverse cholesterol transport from peripheral tissues and from lipid-laden macrophages and arterial smooth muscle cells [28]. HDLC also inhibits atherogenesis through other pathways like alleviating the intimal thickness and atherosclerotic inflammation [29].

Our study reveals that patients with a DBIL/HDLC ratio ≤ 4.45 have significantly better overall survival compared to those with a ratio > 4.45, indicating that the DBIL/HDLC ratio may serve as a simple and effective tool for prognostic assessment. The causes of death in patients with DCM are diverse, including HF and malignant arrhythmias. A high DBIL/HDLC ratio may reflect impaired liver function and is associated with an increased risk of adverse clinical outcomes. In real-world clinical settings, elevated DBIL levels may indicate liver dysfunction, while decreased HDLC levels suggest vascular injury. Therefore, patients with an elevated DBIL/HDLC ratio may benefit from therapies aimed at protecting liver function and vascular health. Additionally, healthcare providers are encouraged to perform more frequent follow-ups and biochemical assessments for these patients. This proactive approach enables clinicians to closely monitor their physiological status and initiate timely interventions in case of any deterioration.

Notably, the results of the subgroup analysis, as shown in Figure 4, reveal a significant interaction between age, the DBIL/HDLC ratio, and long-term mortality. Furthermore, researchers have observed apparent gender differences in DBIL and HDLC levels among the elderly population [30, 31]. Therefore, sex may influence the relationship between the DBIL/HDLC ratio and long-term mortality. Additionally, an increase in the DBIL/HDLC ratio was associated with a higher risk of long-term mortality in both groups.

Consequently, utilizing the DBIL/HDLC ratio as a prognostic marker in DCM provides a comprehensive and objective approach to disease assessment and management. This enables more targeted and effective patient care, ultimately improving outcomes and quality of life in individuals with DCM. However, despite existing supporting evidence, further clinical and mechanistic studies are needed to fully elucidate this relationship and to support the development of preventive and therapeutic strategies for DCM.

## 5. Conclusions

This study uses univariate and multivariate logistic regression to investigate the association of the DBIL/HDLC with clinical outcomes. The DBIL/HDLC ratio could serve as an affordable and simple tool to evaluate prognosis in DCM patients.

## Figures and Tables

**Table 1:** Baseline characteristics of patients with idiopathic DCM according to the long-term clinical outcomes.

	**Patients with NIDCM (** **N** = 986**)**		
**Variables**	**Survival (** **n** = 530**)**	**Death (** **n** = 456**)**	**p** ** value**
Demographic			
Age, years	66.95 ± 5.58	69.77 ± 6.68	< 0.001
Male, *n* (%)	345(65.0%)	299 (65.6%)	0.162
Medical history			
Smoking history, *n* (%)	135 (25.4%)	127 (27.9%)	0.275
Hypertension, *n* (%)	182 (34.3%)	138 (30.3%)	0.967
Diabetes, *n* (%)	125 (23.5%)	120 (26.3%)	0.973
NYHA functional class, *n* (%)			< 0.001
II	134 (25.2%)	103 (22.6%)	
III	201 (37.9%)	185 (40.6%)	
IV	42 (7.9%)	96 (21.1%)	
Parameters on admission			
Serum sodium (mmol/L)	138.88 ± 3.41	137.39 ± 4.35	< 0.001
WBC count (10^9^/L)	7.61 ± 2.77	7.44 ± 2.85	0.370
Neutrophil count (10^9^/L)	5.13 ± 2.69	4.94 ± 2.44	0.260
Lymphocyte count (10^9^/L)	1.61 (1.18–2.10)	1.58 (1.13–2.07)	0.406
Hemoglobin (g/L)	132.00 (122.00–144.00)	131.55 (120.00–144.00)	0.762
Glucose (mmol/L)	6.53 ± 2.77	7.22 ± 3.25	< 0.001
CREA (*μ*mol/L)	90.86 ± 37.22	134.97 ± 97.59	< 0.001
TC (mmol/L)	4.38 (3.65–5.12)	4.00 (3.41–4.90)	0.160
HDLC (mmol/L)	1.08 ± 0.31	0.98 ± 0.32	< 0.001
DBIL (*μ*mol/L)	5.70 ± 11.39	8.48 ± 9.17	< 0.001
Echocardiographic features			
LAD (mm)	42.93 ± 8.83	45.23 ± 8.67	< 0.001
LVEDD (mm)	65.05 ± 8.83	66.53 ± 9.55	0.027
PAP (mmHg)	37.00 (28.00–48.00)	43.00 (33.00–54.00)	0.835
LVEF (%)	35.65 ± 11.65	29.89 ± 9.30	< 0.001

Abbreviations: CREA, creatinine; DBIL, direct bilirubin; HDLC, high-density lipoprotein cholesterol; LAD, left atrial dimension; LVEDD, left ventricular end-diastolic dimension; LVEF, left ventricle ejection fraction; NYHA, New York Heart Association; PAP, pulmonary artery pressure; TC, total cholesterol; WBC, white blood cell.

**Table 2:** Logistic regression analyses for in-hospital mortality.

**Variables**	**Univariate analysis**		**Multivariate analysis**	
	**OR**	**p** ** value**	**OR**	**p** ** value**
D/H score	1.030 (1.017–1.043)	< 0.001	1.019 (1.002–1.035)	0.025
Age	1.046 (0.997–1.098)	0.069		
Males	0.583 (0.272–1.249)	0.165		
Smoke	0.663 (0.333–1.322)	0.244		
Hypertension	1.768 (0.799–3.912)	0.159		
Diabetes	1.009 (0.469–2.168)	0.982		
Serum creatinine	1.008 (1.006–1.011)	< 0.001	1.005 (1.001–1.009)	0.007
lgNT-proBNP	1.000 (1.000–1.000)	< 0.001	1.000 (1.000–1.000)	0.002
Hemoglobin	1.005 (0.987–1.024)	0.571		
Uretic	1.124 (0.390–3.241)	0.829		
LVEF	0.955 (0.919–0.991)	0.016	0.979 (0.936–1.024)	0.360

Abbreviations: D/H, DBIL/HDLC; lgNT-proBNP, log-transformed N-terminal B-type natriuretic peptide; LVEF, left ventricle ejection fraction; OR, odds ratio.

**Table 3:** Logistic regression analyses for long-term mortality.

**Variables**	**Univariate analysis**		**Multivariate analysis**	
	**OR**	**p** ** value**	**OR**	**p** ** value**
D/H score	1.049 (1.031–1.067)	< 0.001	1.026 (1.005–1.048)	0.016
Age	1.077 (1.055–1.101)	< 0.001	1.064 (1.034–1.094)	< 0.001
Males	0.971 (0.747–1.263)	0.827		
Smoke	0.903 (0.681–1.198)	0.479		
Hypertension	1.195 (0.914–1.563)	0.193		
Diabetes	0.855 (0.640–1.142)	0.290		
Serum creatinine	1.017 (1.014–1.021)	< 0.001	1.017 (1.012–1.022)	< 0.001
lgNT-proBNP	1.000 (1.000–1.000)	< 0.001	1.000 (1.000–1.000)	0.483
Hemoglobin	0.996 (0.990–1.003)	0.305		
Uretic	0.559 (0.358–0.873)	0.011	0.541 (0.278–1.054)	0.071
LVEF	0.948 (0.934–0.962)	< 0.001	0.951 (0.934–0.968)	< 0.001

Abbreviations: D/H, DBIL/HDLC; lgNT-proBNP, log-transformed N-terminal B-type natriuretic peptide; LVEF, left ventricle ejection fraction; OR, odds ratio.

**Table 4:** Multivariate Cox proportional hazard regression models for long-term mortality.

**Variables**	**Univariate analysis**		**Multivariate analysis**	
	**HR (95% CI)**	**p** ** value**	**HR (95% CI)**	**p** ** value**
D/H score	1.005 (1.004–1.007)	< 0.001	1.011 (1.005–1.018)	< 0.001
Age	1.053 (1.038–1.068)	< 0.001	1.043 (1.025–1.061)	< 0.001
Males	0.931 (0.768–1.130)	0.469		
Smoke	1.099 (0.896–1.350)	0.365		
Hypertension	0.906 (0.742–1.107)	0.335		
Diabetes	1.273 (1.033–1.568)	0.030	1.366 (1.077–1.732)	0.010
lgNT-proBNP	1.000 (1.000–1.000)	< 0.001	1.000 (1.000–1.000)	< 0.001
Hemoglobin	0.996 (0.991–1.001)	0.124		
Uretic	1.780 (1.242–2.551)	0.002	1.655 (1.030–2.659)	0.037
LVEF	0.964 (0.954–0.975)	< 0.001	0.974 (0.962–0.985)	< 0.001

Abbreviations: D/H, DBIL/HDLC; lgNT-proBNP, log-transformed N-terminal B-type natriuretic peptide; LVEF, left ventricle ejection fraction; OR, odds ratio.

## Data Availability

The data generated or analyzed during this study are not publicly available. Authors elect to not share data.
